# Grain-Scale Anisotropic Analysis of Steady-State Creep in Oligocrystalline SAC Solder Joints

**DOI:** 10.3390/ma14205973

**Published:** 2021-10-11

**Authors:** Qian Jiang, Abhishek Nitin Deshpande, Abhijit Dasgupta

**Affiliations:** Center for Advanced Life Cycle Engineering (CALCE), Mechanical Engineering Department, University of Maryland, College Park, MD 20742, USA; despande@umd.edu

**Keywords:** lead-free solder, anisotropic steady-state creep, Hill–Norton model, coarse-grained finite element analysis, grain boundary creep, cohesive element

## Abstract

Heterogeneous integration is leading to unprecedented miniaturization of solder joints, often with thousands of joints within a single package. The thermomechanical behavior of such SAC solder joints is critically important to assembly performance and reliability, but can be difficult to predict due to the significant joint-to-joint variability caused by the stochastic variability of the arrangement of a few highly-anisotropic grains in each joint. This study relies on grain-scale testing to characterize the mechanical behavior of such oligocrystalline solder joints, while a grain-scale modeling approach has been developed to assess the effect of microstructure that lacks statistical homogeneity. The contribution of the grain boundaries is modeled with isotropic cohesive elements and identified by an inverse iterative method that extracts material properties by comparing simulation with experimental measurements. The properties are extracted from the results of one test and validated by verifying reasonable agreement with test results from a different specimen. Equivalent creep strain heterogeneity within the same specimen and between different specimens are compared to assess typical variability due to the variability of microstructure.

## 1. Introduction

Sn-Ag-Cu (SAC) solder alloys are widely used in microelectronic packaging to provide mechanical, electrical and thermal connections between different structures/materials for proper function. Their behavior and failure mechanisms are critical to the reliability of integrated packaging systems. The development of heterogeneous integration has led to solder interconnects located in complex structures such as three-dimensional wafer-level packages, multi-die stacks, etc., and subject to quite demanding loading conditions [1,2]. Appropriate material properties are necessary for numerical simulations in order to accurately assess their performance and reliability within the system. Extensive studies have been conducted to characterize the mechanical properties of SAC alloys; however, the results vary not only between groups but also between multiple measurements of the same group [3,4]. This is usually attributed to their stochastic microstructure, which typically consists of only a few highly anisotropic grains, in either discrete or interlaced morphology [5,6,7,8]. Moreover, the miniaturization of packages leads to an increase in the number of I/Os and a decrease in pitch size, which also reduces the size and volume of individual solder joints. The solder joint may be of similar length scale to the grain size, in microbumps. In view of this fact, both testing and modeling schemes should be based on such representative microstructures.

Crystal plasticity finite element (CPFE) models have been widely used to simulate anisotropic deformation of SAC solder joints at the grain scale, based on dislocations moving on preferred slip systems in accordance with phenomenological flow models [9,10]. To simulate the intergranular fracture observed in tin-based solder joints [11,12], the CPFE model is equipped with a cohesive zone model, in which the crack propagation is described by a traction-separation relationship [13]. Another modeling strategy emphasizes the heterogeneous microstructure inside SAC joints. The effect of morphometrics of submicron intermetallic compounds on mechanical response is investigated by multiscale modeling methods, where three-dimensional representative volume elements (RVEs) were imported directly from tomographic images [14,15]. Xu et al. incorporates the multi-scale modeling strategy into CPFE, by modeling the SAC grains as particle matrix composites [16]. Fully representative models of the directionally solidified samples were developed based on the microstructures achieved at different loading rates, and compared with experimental results. However, the above-mentioned modeling approaches that incorporate anisotropic properties or/and microstructural features may be computationally expensive to some extent.

This paper focuses on the viscoplastic behavior of grain boundaries, an indispensable element in the grain-scale modeling of SAC solder joint. Since the steady-state creep at grain boundaries is currently difficult to measure directly and the complex mechanisms that dominate creep at grain boundaries remain unclear, its viscoplastic properties are determined here by matching simulation with experimental results using an inverse engineering approach [17]. Comparison of equivalent creep strain fields shows significant differences between samples, even under similar loading states. The proposed grain-scale model, including anisotropic grains and isotropic grain boundaries, provides insight into the effect of grain structure on the steady-state creep of the joint under given conditions. Considering the stochastic nature of grain morphology in oligocrystalline SAC solder interconnects, grain-scale analysis can provide a valuable tool for parametric studies of the resulting stochastic variability of solder joint creep response. Such studies are also valuable for determining the best- and worst-case grain morphologies. Such a simulation-aided virtual testing capability allows for efficient assessment of stress/strain severity, and helps reduce the time and cost of physical testing, when assessing stochastic design margins and reliability guidelines under various types of life-cycle loading conditions.

## 2. Multigrain TMM Specimen

**Figure 1:**
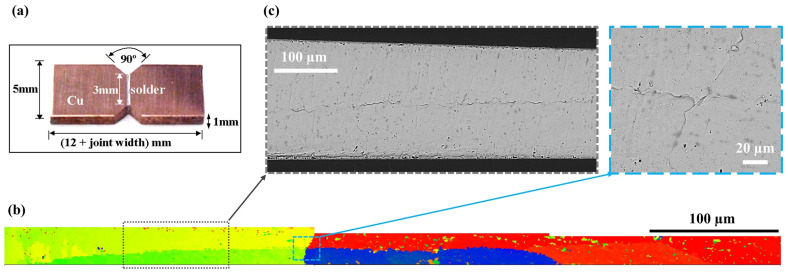
Sample 1 used in the TMM shear test: (**a**) nominal dimensions of modified Iosipescu shear specimens, (**b**) EBSD scanning of the solder joint of Sample 1, (**c**) ESEM images of failure patterns along the grain boundary after creep test.

A customized thermomechanical microscale (TMM) system was developed for mechanical testing of specimens with functional joint dimensions and similar coarse-grained microstructures, with the aim of minimizing the influence of factors other than grain configurations. Comprehensive details on test setup and specimen design are provided elsewhere [18,19,20]. In the current study, creep tests were performed on modified lap-shear specimens consisting of Sn-3Ag-0.5Cu (wt.%) (SAC305) solder joint and two copper platens without metallization layers. Figure 1a shows a sample specimen [21,22] with nominal dimensions. The specimen is nominally deformed in shear, and the 90-degree notches on both sides facilitate uniform stress distribution. The stress state of each specimen is evaluated individually, depending on the applied force and joint dimensions. Such TMM specimens usually contain only one grain, or at most a few grains and are referred to as grain-scale specimens, since the length scale of the test specimen is similar to that of individual grains. As an example, Figure 1b provides an EBSD image of Sample 1, which contains four crystals. The failure patterns observed after testing (Figure 1c) indicates severe deformation discontinuity near the grain boundaries and crack propagation along the interfaces between crystals.

Grain-scale testing has been used to characterize solder properties, to gain insight into possible variabilities between specimens, and to study the effects of microstructure. Further, the test results help to understand the influence of microstructure and its correlation with mechanical response and variability, through a grain-scale modeling methodology.

## 3. Modeling Strategy

The coarse-grain microstructure, in conjunction with the anisotropy of body-centered tetragonal (BCT) β-Sn, renders each individual microbump mechanically unique. Thus, each joint will demonstrate unique stress-strain response. A grain-scale modeling strategy is proposed to evaluate the degree of variation between joints due to microstructure at a given condition, which is achieved by the anisotropic properties of individual grains and the contribution of grain boundaries. The typical microstructure of a coarse-grained SAC solder joint is divided into multiple tiers depending on the critical length scales. Tier 3 represents a general solder joint containing only one or at most a few grains, the so-called oligocrystalline microstructure, while Tiers 0–2 are within a single grain. The modeling approach for single crystals and grain boundaries is presented in Section 3.1 and Section 3.2, respectively. Calibration of the single crystal secondary creep behavior was presented in an earlier paper [23]. Calibration of the grain boundary model via their application to oligocrystalline test specimens (Tier 3) is illustrated in Section 4.

### 3.1. Anisotropic Single SAC Crystal: Crystal Viscoplasticity Model and Hill–Norton Continuum Model

A hierarchical multiscale crystal viscoplasticity (CV) model has been proposed by the authors to capture the anisotropic steady-state creep behavior of single SAC crystal, based on key microstructure features (including Tier 2 and below) and dislocation motion [23]. The main concepts of that modeling approach are briefly reviewed here for completeness. The deformation mechanisms based on important microstructural characteristics and features at pertinent length scales is modeled using appropriate mechanisms, and then the key variables are transferred from one level to the next higher one, as the necessary basic building blocks. Tier 2 represents a single SAC crystal in which the dendritic structure of Sn is surrounded by a eutectic Ag-Sn mixture. Since the applied load is shared between these two phases, Mori-Tanaka homogenization [24] is performed to estimate the mean-field viscoplastic behavior. Tier 1 focuses on the eutectic mixture, where the statistically uniform distribution of nanoscale Ag_3_Sn particles in the *β*-Sn matrix leads to dispersion hardening. The mobile dislocations are pinned or retarded by the dispersed precipitates, corresponding to the enhanced macroscopic creep resistance. Based on microstructural analysis and experimental results, the bypass and recovery process for dislocations to recover from obstructions is modeled as a combination of dislocation climb and detachment mechanisms that dominate in the low- and high-stress regions, respectively. Tier 0 is the smallest length scale considered in the CV model and represents the anisotropic steady-state creep of the Sn phase. The presence of multiple slip systems [25] renders the movement of dislocations along facile directions and the overall creep behavior highly dependent on the given loading conditions and grain orientation. Two creep activation energies were measured in earlier experimental studies on single-crystal Sn specimens, suggesting the existence of two competing mechanisms [26,27,28]. Thus, the steady-state creep of a single *β*-Sn crystal is modeled as dislocation climb and slip acting in conjunction [17]. 

This semianalytical model, calibrated on length scales for which experimental results are available (single crystal *β*-Sn and SAC specimens), explicitly estimates the steady-state strain rate based on a given stress state, temperature, and key microstructural features. Although this model can be directly implemented for numerical analysis, an alternative approach is proposed in order to perform computationally efficient finite element simulations in microelectronic assemblies. The Hill quadratic criterion provides a user-friendly approach to characterize anisotropic behavior, analogous to the von Mises criterion used for isotropic materials. The Hill effective stress $\sigma_{Hill}$ under multiaxial stress state is defined as:

(1) $$\sigma_{Hill}=\sqrt{F{\left(\sigma_{22}-\sigma_{33}\right)}^{2}+G{\left(\sigma_{33}-\sigma_{11}\right)}^{2}+H{\left(\sigma_{11}-\sigma_{22}\right)}^{2}+2L\sigma_{23}^{2}+2M\sigma_{13}^{2}+2N\sigma_{12}^{2}}$$

where F, G, H, L, M and N are parameters that elucidate the anisotropic state, and are usually obtained experimentally at specific load directions. In isotropic materials, all of these constants default to the value of 1, and thus Hill equivalent stress becomes the same as von Mises’ equivalent stress in that case. The CV model described above can serve as a virtual test method to predict the strain rate under selected simple stress conditions, thus providing a method to estimate the values of the Hill constants. The subscripts 1, 2 and 3 correspond to the crystal main directions [100] [010] and [001], respectively. Therefore, SAC crystals can be simplified to an anisotropic homogeneous material in numerical simulations. The corresponding strain rate will take the following form

(2) $$\dot{\varepsilon }=\frac{\partial \sigma_{Hill}}{\partial \sigma }{\dot{\varepsilon }}_{eq}={\dot{\varepsilon }}_{eq}\cdot \frac{1}{\sigma_{Hill}}\;\left[\begin{array}{c} G\left(\sigma_{11}-\sigma_{33}\right)+H\left(\sigma_{11}-\sigma_{22}\right)\\ F\left(\sigma_{22}-\sigma_{33}\right)+H\left(\sigma_{22}-\sigma_{11}\right)\\ G\left(\sigma_{33}-\sigma_{11}\right)+F\left(\sigma_{33}-\sigma_{22}\right)\\ 2N\sigma_{12}\\ 2M\sigma_{31}\\ 2L\sigma_{23} \end{array}\right],$$

where $\dot{\varepsilon }$ and σ represent the strain rate and stress state, respectively, and are written in Voigt notation as 6-by-1 vectors. ${\dot{\varepsilon }}_{eq}$ is the Hill equivalent strain rate. It is a scalar multiplier that depends on the governing flow rule.

**Figure 2:**
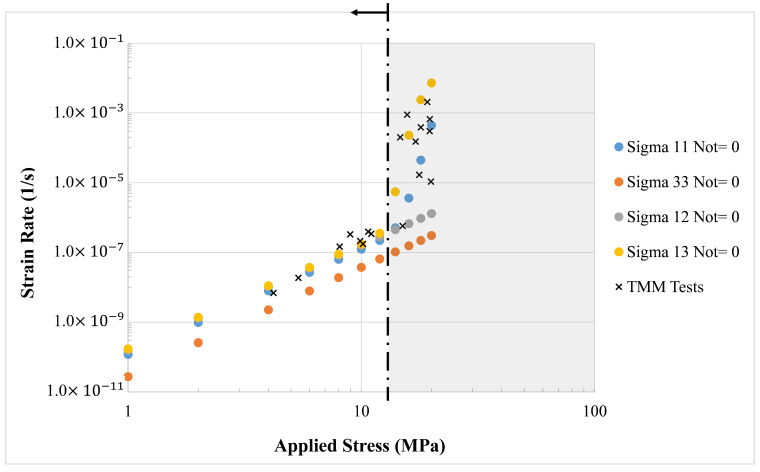
Strain rates along the loading directions calculated from the CV model.

Figure 2 displays the strain rates along loading directions predicted by the CV model for selected uniaxial or pure shear loading conditions at room temperature. For example, “Sigma 11 Not= 0” means that all stress components except $\sigma_{11}$ are equal to zero, that is, the uniaxial stress state. As modeled, creep is dominated by dislocation climb at low stresses and then shifts to be dominated by dislocation detachment mechanism as the stress increases. The dominance of these two mechanisms can be clearly discerned from the sudden increase in the stress exponent in Figure 2. Although different stress states start to be governed by the dislocation detachment mechanism at different values, which are closely related to the slip systems activated, all stress states are dominated by the dislocation climb mechanism at stresses below 12 MPa. Thus, the steady-state creep response in the low stress region can be expressed by Norton’s power law.

(3) $${\dot{\varepsilon }}_{eq}=A{\left(\sigma_{Hill}\right)}^{n}$$

**Figure 3:**
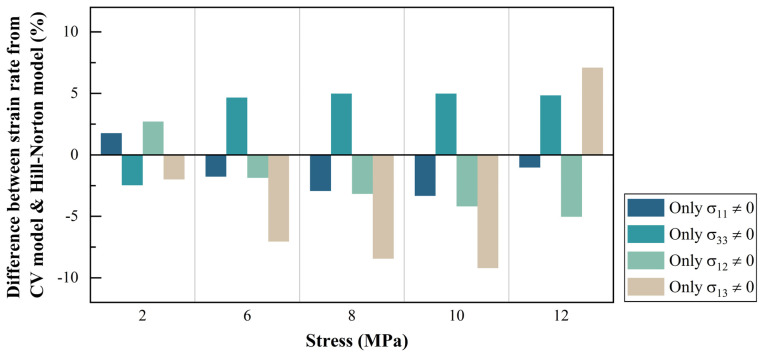
Percentage difference in strain rates along the loading directions obtained from the CV model and Hill–Norton formula in the low stress region.

Assuming that all stress conditions share an identical stress exponent at a constant temperature, the Hill–Norton parameters are extracted for low stresses (<12 MPa) from the virtual test results in Figure 2 and summarized in Table 1. Due to crystal symmetry, the crystallographic directions [100] and [010] are almost equivalent to each other, so that F=G and L=M. F is set equal to one, and all other Hill’s anisotropic constants are normalized to F. The quality of this fit is shown in Figure 3 where strain rate components estimated from the best-fit Hill–Norton formula are compared with those obtained from the CV model.

### 3.2. Grain Boundary

Creep in polycrystalline solids is a complex process involving severe deformation of grains and accommodation of grain boundaries. Although the creep mechanisms of individual grains have been thoroughly investigated and various models have been proposed to explain that, similar studies on the viscoplastic properties of grain boundaries are still somewhat limited. A unified constitutive model is usually used to describe the overall behavior of polycrystalline materials without distinguishing the respective contributions of grains and grain boundaries. The mechanism behind this constitutive model may be diffusion or dislocation movement, either within the crystal or near the boundary interfaces [29,30,31]. This leaves a dilemma for coarse-grained materials, where neither the deformation of the grains themselves nor the deformation of the grain boundaries can be ignored. In order to quantify their viscoplastic properties, in this work, grain boundaries are modeled as isotropic materials and implemented in numerical simulations for comparison with tests.

An analytical creep model was developed [32] on the basis of dislocation climb at grain boundaries, in which shearing occurs along slip bands blocked by the grain boundaries. After a quantitative comparison with creep data of pure Sn and Sn-based alloys, the model is given in the following form:

(4) $$\dot{\varepsilon }/D_{GB}\propto \left(\frac{50\delta }{b^{3}}\right)\left(\frac{\sigma b^{3}}{kT}\right)\left(\frac{Lb^{3}}{\lambda^{4}}\right)$$

where δ is the grain boundary thickness, usually on the scale of atomic size. L is the grain size, λ is the slip band spacing, and b is the magnitude of the Burgers vector. This model indicates that the strain rate is linearly dependent on the stress at a given temperature. A similar linear relationship was used by researchers to model the relative displacement rates of grains meeting at boundaries, in the numerical analysis of nonuniform flow in polycrystals, which are attributed to the grain boundary sliding and viscosity inside the crystals [33].

(5) $${\dot{u}}_{GB}=\left(\frac{\delta }{\eta_{B}}\right)\tau ,$$

${\dot{u}}_{GB}$ represents the displacement rate; $\eta_{B}$ denotes the viscosity of grain boundary, which depends on the boundary configuration and is proportional to the temperature. Although the real grain boundary is neither flat nor uniform, it is simplified as a homogeneous isotropic material in current work, for the purpose of implementation in numerical simulations. Motivated by Equations (4) and (5), the steady-state creep rate in this study is assumed to follow a linear Newtonian isotropic viscous model, given by

(6) $${\dot{\varepsilon }}_{GB}=A_{GB}\sigma ,$$

where $A_{GB}$ is a unified parameter that represents the combined consequence of multiple factors, such as boundary configuration, thickness, diffusivity, temperature, etc. The value of $A_{GB}$ is determined by calibrating the simulation with experimental result.

## 4. Numerical Simulations

The coarse-grained TMM specimens are constructed in FEM based on the grain orientations obtained from the EBSD surface scanning. The effect of the grain boundaries is modeled by an ultrathin layer of cohesive elements whose viscoplasticity is identified from the inverse finite element method. Section 4.1 presents the determination and validation process. A tricrystal BGA joint with assumed grain orientation and morphology is modeled in Section 4.2 using the extracted grain boundary properties, as a simplified example of the grain-scale modeling approach. Further analysis of the simulation results is presented subsequently.

### 4.1. Calibration and Validation of Grain Boundary Properties on TMM Specimens

**Figure 4:**
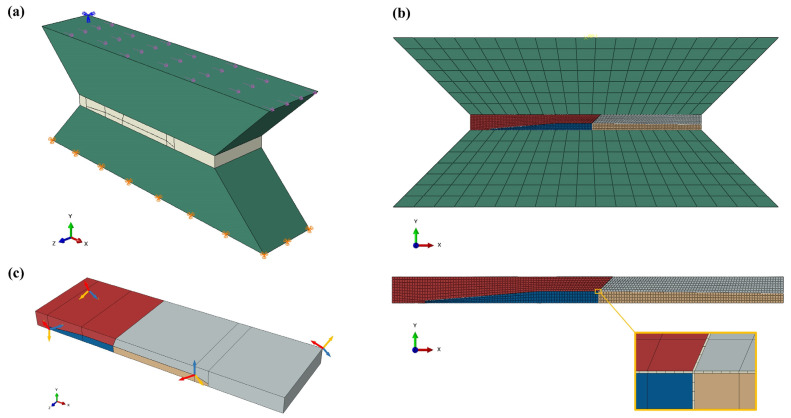
FEA model of TMM shear specimen: (**a**) boundary and loading conditions, (**b**) mesh and (**c**) local coordinates indicating the direction of each grain of Sample 1.

Figure 4a shows the model construction of the middle region of the TMM specimen (loaded region spanning across the two loading grips), where the solder joint is connected to two copper platens (in green). The bottom surface is fully constrained, and a uniform traction stress is applied horizontally on the top surface, so as to mimic the shear loading and boundary conditions applied in the physical shear tests. Previous studies examined both surfaces of TMM solder joints by cross-polarization microscopy and detected that the number and configuration of grains were almost invariant over the entire thickness of the specimen [21]. Similar results have been observed by other groups through orientation imaging microscopy (OIM) [34]. Therefore, the grain orientation from the surface electron backscatter diffraction (EBSD) scan is considered to be representative of the entire volume of the joint. Sample 1 presented in Figure 1 [21,22] is used for calibration and the grain structure is modeled accordingly (Figure 4b). Approximate creep deformation measurements initially reported in the literature were obtained with a linear variable differential transformer (LVDT) [18]. In the current work, the resulting solder joint strain estimates have been improved, based on additional studies that provide correlation between LVDT readings and solder strain, using an in situ digital image correlation (DIC) virtual extensometer [20]. There are three main grains in this specimen, separated by grain boundaries. There are two coordinate systems: the global system $\left\{X,\;Y,\;Z\right\}$ is intended to indicate the loading direction and orientation relative to specimen geometry, while the local coordinate system defines the orientation of the individual grains, where the axes $\left\{a,\;b,\;c\right\}$ correspond to the crystal principal directions $\left\{\left[100\right],\;\left[010\right],\;\left[001\right]\right\}$. Figure 4c depicts the grain orientations of Sample 1.

Each SAC grain is modeled as an anisotropic viscoplastic material, using the Hill–Norton model described in Section 3.1 and shown in Figure 2 and Equation (3). The experimentally calibrated Hill–Norton model constants used here are from earlier work of the authors [23] and are given in Table 1 for completeness. 

The grain boundary region is discretized by a monolayer of cohesive elements with continuum macroscopic properties. Cohesive elements are a set of elements implemented in ABAQUS to model bonding, adhesive interfaces, fractures, or similar configurations that can be thick or infinitely thin. The constitutive response of cohesive elements can be defined by a continuum approach or by a traction-separation description [35,36]. Following methods recommended in the literature, the grain boundary layer is approximated in the numerical model as a flat cohesive zone with nominal thickness of 1 µm. This is understood to be a numerical approximation for computational expedience, rather than the actual physical thickness of the grain boundary [33]. The 3D cohesive element formulation used in this commercial finite element analysis is capable of only three strain components defined in the local tangential-normal coordinate system: one extensional strain component in the thickness direction (along the normal to the grain-boundary surface) and two transverse shear strain components in orthogonal planes; all other strain components are assumed to be zero for the constitutive calculations. This deformation mechanism corresponds to the decohesion and sliding of grain boundaries.

**Figure 5:**
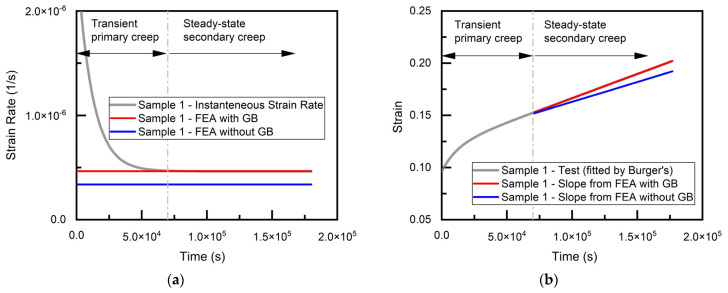
Simulation results of Sample 1 after calibration (**a**) strain rate (**b**) strain.

The nominal strain rate is estimated by the same procedure as in the experiments, namely, the average displacement rate along the loading direction (X) divided by the initial height of the joint, $\dot{\gamma }=\frac{{\dot{u}}_{x}}{h}$. The values of $A_{GB}$ are adjusted until the simulated strain rate matches the measurement. The result after calibration ($A_{GB}=1.05E-6$ at room temperature) is plotted against the instantaneous strain rate from the experiment, as shown in Figure 5a. For comparison, strain rate without the contribution of grain boundaries is also provided, where the grain boundary is modeled as a perfectly elastic material with no viscoplasticity. For calibration purposes, experimental data of strain versus time, including primary and secondary creep regions, were fitted with the four-element Burgers model [37,38],

(7) $$\varepsilon \left(t\right)=k_{0}+k_{1}t+k_{2}\left(1-e^{-k_{3}t}\right),$$

The instantaneous strain rate was determined as the time derivative of the fitted model. The strains are plotted versus time in Figure 5b, according to the constant rates from the steady-state finite element simulations, and compared with the near steady-state region of the experimental data. 

**Figure 6:**
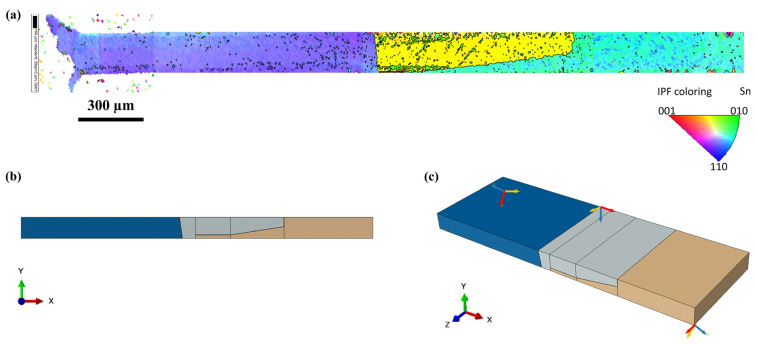
Grain structure of Sample 2 [21,22]: (**a**) EBSD image, (**b**) grain structure in FEA, (**c**) local coordinates indicating the direction of each grain of Sample 2.

**Figure 7:**
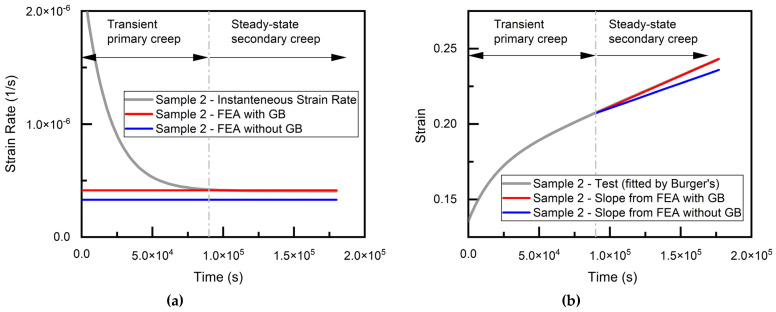
Simulation results of Sample 2 (**a**) strain rate (**b**) strain.

As discussed above, this grain boundary model is next verified by modeling a second shear test specimen and comparing the prediction with experimental results adapted from the literature [21], using the same DIC correlation, as discussed above for Sample 1. The grain orientation of the coarse-grained Sample 2 was obtained from EBSD (Figure 6a). A FEA model similar to that of Sample 1 was constructed for Sample 2, where the grain structure of the joint is shown in Figure 6. Considering the possible issue of mesh density sensitivity [39], which arises from stress singularities, the same sizing control as that of Sample 1 was performed for the Cu pads, SAC grains and grain boundaries, respectively, for mesh seed assignment. Since both tests were conducted at room temperature, $A_{GB}$ is assumed to be identical for Samples 1 and 2. Therefore, the simulation of Sample 2 is performed with the calibrated parameter $A_{GB}$ value obtained from Sample 1. Both strain rate and strain history are compared with experimental results, as illustrated in Figure 7. This output of FEA shows good agreement with the experiment (about 2% higher).

**Figure 8:**
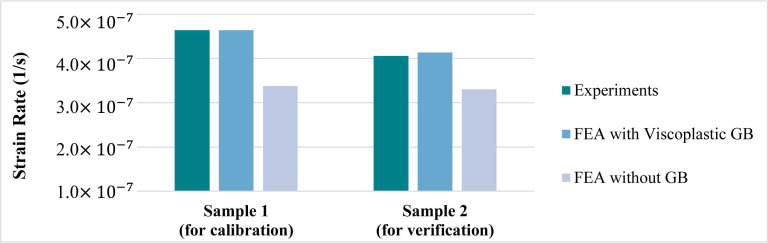
Comparison of experiments and simulations for TMM Samples 1 and 2.

The strain rates of Samples 1 and 2, from experiments and simulations, with and without the contribution of viscoplastic grain boundaries, are summarized in Figure 8. Although the volume fraction of grain boundaries is rather small compared to the entire joint, their influences on the time-dependent inelastic deformation is non-trivial. The incorporation of grain boundary regions with isotropic viscoplasticity in the finite element analysis increases the overall strain rate of Samples 1 and 2 by about 37% and 26%, respectively. Based on this result, both the deformation within individual grains and the accommodation function of grain boundaries should be considered in appropriate analysis of coarse-grained SAC joints.

**Figure 9:**
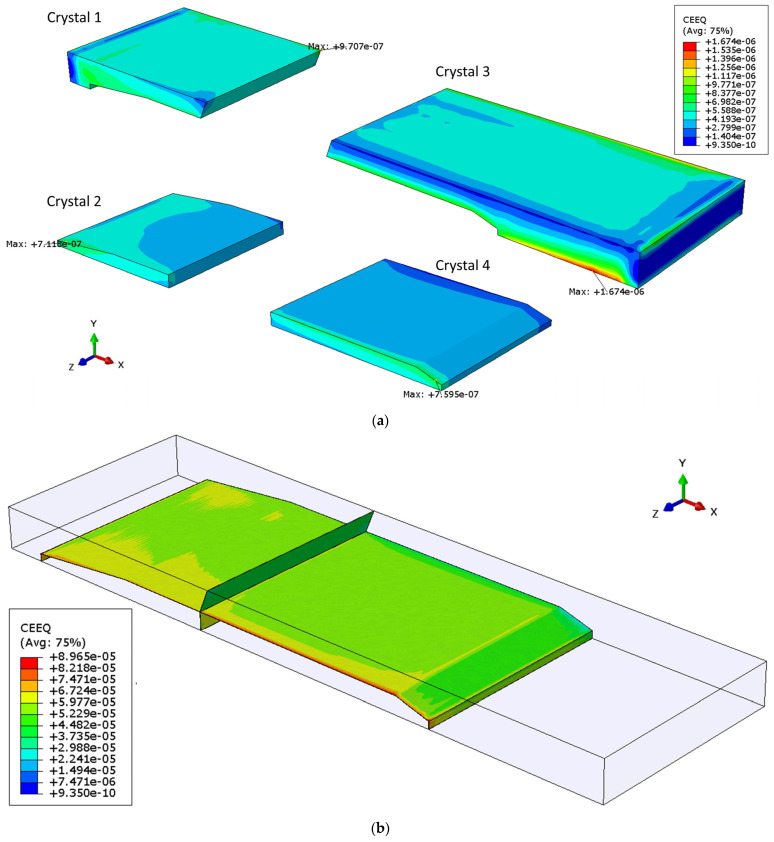
Equivalent creep strain contour of Sample 1 in: (**a**) each crystal, (**b**) grain boundary region.

Contours of the equivalent creep strains for Sample 1 are provided in Figure 9. The equivalent creep strain in the grain boundary region is much higher than that in each individual grain, with a ratio of more than 50 between the maximum values. This would explain, to some extent, the observed distinct damage pattern along the grain boundaries, reported after the shear test, and shown in Figure 1 [21,22]. The maximum strain for all crystals is located at the edge of Crystal 4, which is connected to the clamped Cu platen. The maximum strains in both Crystals 2 and 4 are at the edges adjacent to the cohesive elements, where they meet neighboring crystals. The presence of grain boundaries accommodates the strong mismatch between grains through limited normal traction and intensive sliding. In Crystal 3, the strain is concentrated at one vertex, which is actually the intersection point of Crystals 1, 3 and the upper Cu platen.

**Figure 10:**
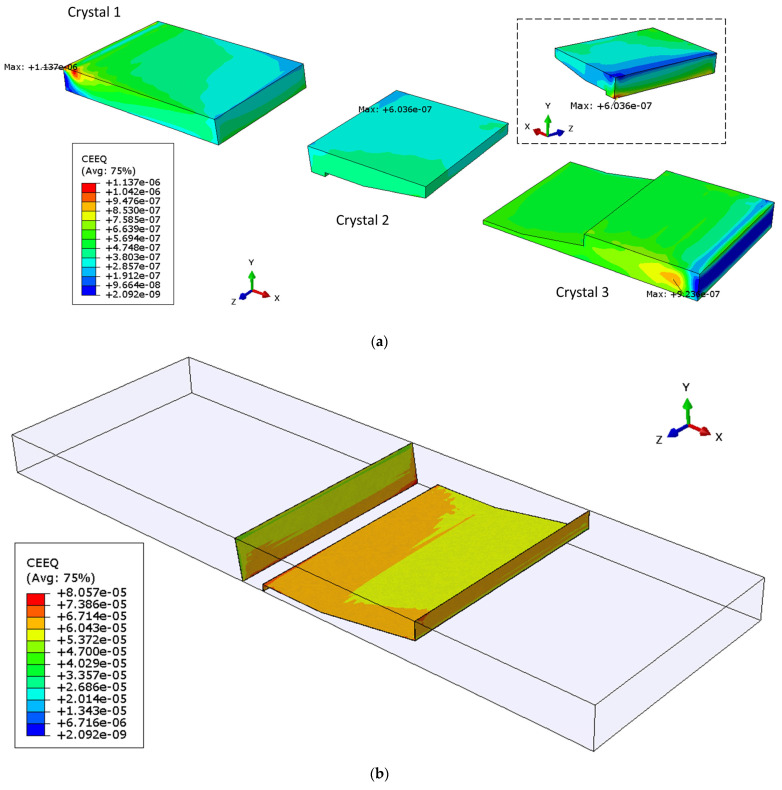
Equivalent creep strain contour of Sample 2 in: (**a**) each crystal, (**b**) grain boundary region.

Figure 10 displays the corresponding contours of the equivalent creep strain for Sample 2. The maximum strain ratio between the grain boundary and crystals is more than 70. The creep strain of Crystal 1 is concentrated on the side surface and near the corner of the joint. The maximum value for Crystal 2 is located at one vertex common to Crystals 1, 2 and the bottom copper platen. In Crystal 3, the location of the peak strain is inside the grain.

Within each joint, the concentration of creep stain varies considerably from crystal to crystal, which can be attributed to its own grain orientation, the misorientation with adjacent grains, the behavior of grain boundaries and the consequent complex stress distribution, etc. This strain heterogeneity is to some extent consistent with that observed in tested failed samples: the solder joint may crack along the interfacial layer connected to other structures/materials, or through the interior of the bump. In addition, internal cracks can be either transgranular, i.e., across grains, or intergranular, i.e., decohesion or sliding along grain boundaries.

Although the nominal far-field shear stress applied to Sample 1 is slightly lower than that of Sample 2, the creep strain field within it is much more severe. The maximum equivalent creep strain at crystals and grain boundaries for Sample 1 was 47% and 11% higher, respectively, than corresponding values in Sample 2. Stress concentration is thought to be closely related to the formation of critical subgrains, the stimulation of recrystallization, and eventually the initiation of microcracks. Since the viscoplastic deformation of oligocrystalline solder joints is a rather complex phenomenon, several microstructural features, such as grain morphology (size and orientation), grain boundary distribution; external loading states, should be considered in order to better understand and access their thermomechanical response.

### 4.2. Grain-Scale Analysis of a Tri-Crystal BGA Joint

**Figure 11:**
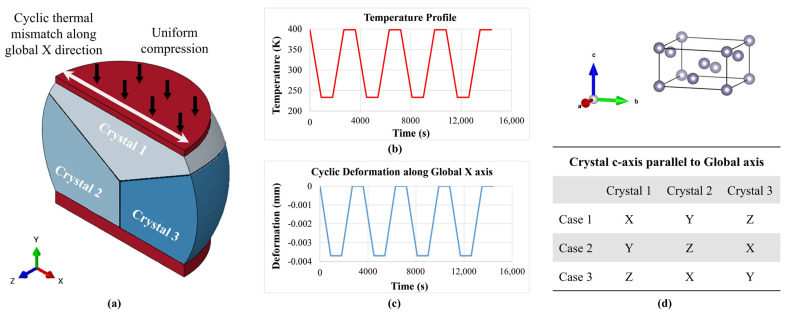
Typical tricrystal BGA joint: (**a**) geometry and loading condition; (**b**) temperature profile; (**c**) cyclic horizontal deformation and (**d**) three combinations of crystal orientations.

Experimental observations of oligocrystalline BGA grain morphology reveal a wide range of typical grain morphologies, ranging from single-crystal joints to beach-ball structures. In this study a simple representative example of a tricrystalline PWA solder joint is chosen for illustrative purposes. Grain-scale creep analysis is conducted to demonstrate the insights possible for oligocrystalline solder joints. The solder joints located at the diagonal corner of BGA packages (or corner of the die shadow in some cases) are commonly considered ‘critical’ because they are subject to the largest deformation caused by the thermal expansion mismatch between the package and the PCB. A critical joint in such position is used for illustrative grain-scale analysis in this section. In addition to thermal cycling, this entire package is also subjected to constant out-of-plane compression by an upper leaf spring, as is often done to provide a stable thermal connection for packages with heat sinks. Figure 11a depicts the geometry and grain structure of the solder joint. Since the purpose of this simplified illustrative example is to focus on the utility of grain-scale analysis, other complexities such as warpage induced out-of-plane deformations are ignored here. The reference baseline morphology is assumed to contain three mutually orthogonal crystals, each with its principal axes aligned with the global coordinate axes. Parametric studies of the effect of grain orientation are conducted by systematically varying the grain orientations. A layer of cohesive elements with nominal model thickness of 1 μm is modeled to represent the presence of grain boundaries. The grain boundary thickness is assumed to be the same as that in the calibration models used above in Figure 4 and Figure 6. The top and bottom faces of this solder joint are attached to Cu pads. The interfacial Cu-Sn intermetallic compound (IMC) layers are ignored for model simplification. As a simplification of the loading conditions, the vertical compression is represented by a constant pressure uniformly distributed on the top Cu pad, while the thermal expansion mismatch due to temperature cycling is approximated as a cyclic horizontal relative displacement between the top and bottom copper pads. The temperature and displacement profile are provided in Figure 11b,c. Due to symmetry, only half of the joint was modeled to improve computational efficiency. As shown in Figure 11d, three combinations of grain orientations were simulated for comparison.

**Figure 12:**
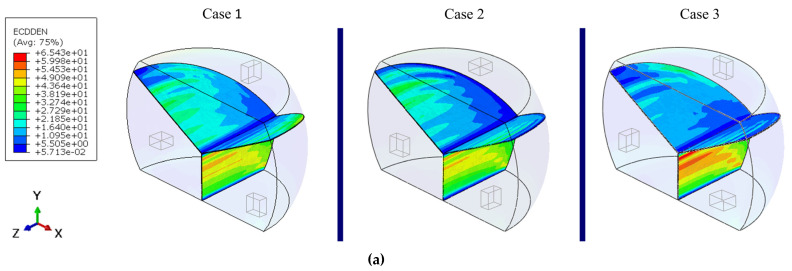

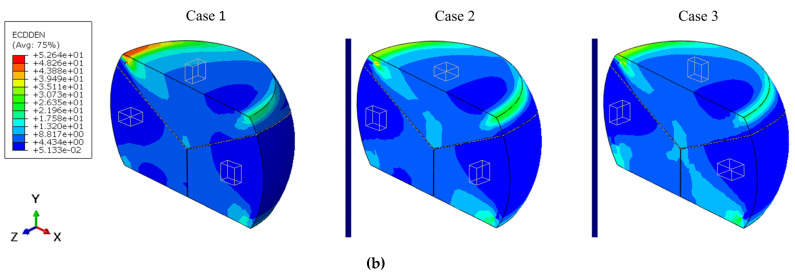
Contour plot of the creep work density dissipated at the end of the fourth cycle in the (**a**) grain boundaries (cohesive elements), and (**b**) grains.

Figure 12 provides the contour of the creep work density dissipated at the grain boundaries at the end of fourth cycle. In all cases, the energy dissipated at the boundary between Crystals 2 and 3 is always significantly higher than at the other two boundary segments. This is due to the fact that one of the main loads is the steady compression along the global Y-direction, which is parallel to the boundary between Crystals 2 and 3. Given the limited thickness of the grain boundary, the out-of-plane tensile deformation is negligible compared to the lateral shear deformation. Among all modeled cases, Case 2 dissipates the least creep work density at the 2–3 boundary, because the creep resistance of Crystals 2 and 3 is the same along the Y direction, thereby minimizing the mismatch along the compressive load path. Likewise, Case 3 exhibits an overall higher creep work density dissipation because the creep resistance along the loading directions, either vertical compression or horizontal cyclic deformation, is different between Crystals 2 and 3.

**Figure 13:**
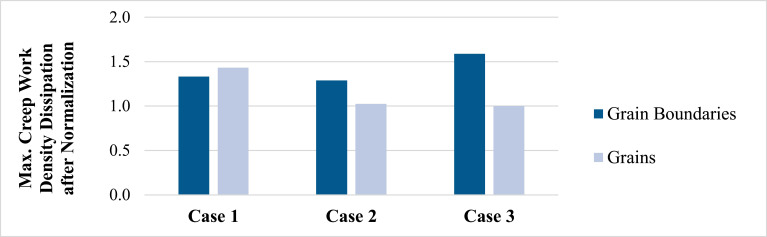
Normalized max. creep work density dissipated at the end of the fourth cycle.

Figure 13 summarizes the normalized values of the maximum creep work density dissipated by the grain boundaries and grains, respectively, at the end of the fourth cycle. These results are normalized by the smallest obtained value, that is, the grains in Case 3. The location of the maximum creep work dissipation remains constant in all cases, i.e., in the case of grain boundaries, between Crystals 2 and 3 (Figure 11a); in the case of grains, at the interface between Crystal 1 and the upper Cu pad (Figure 11b). This interfacial mismatch strain is likely to be even higher in reality, since the interfacial intermetallic layer was ignored in this simplified illustrative example. There is significant variation in the creep magnitudes between the different cases considered. The differences between the three cases are up to 23.2% and 43.2% for creep work density in grains and grain boundaries, respectively. Simple thermomechanical power-law fatigue models in the literature [40] suggest that this difference in creep dissipation can translate to a corresponding variation of up to 70–85% in temperature cycling fatigue durability.

For individual cases, there is a maximum difference of 58.8% in the creep between grains and grain boundaries. While the overall creep work density dissipation at the grain boundaries is higher than that within the grains, the maximum value is not necessarily located at the grain boundaries. As an example, in Case 1, the maximum dissipated creep work density within the grain is 8% higher than that in the grain boundaries. This explains, to some extent, the observed piece-to-piece variability in the damage modes of solder joints between transgranular and intergranular damage. Even for a specific joint with a fixed grain shape and under a constant loading condition, the location and dissipation of the maximum creep work density can vary considerably with grain orientations.

## 5. Conclusions

The purpose of this paper is to develop the capability for grain-scale viscoplastic numerical modeling of polycrystal SAC solder joints, by providing the contribution of grain boundaries to the steady-state creep of coarse-grained SAC solder joints by an inverse fitting approach. Although SAC interconnects are usually modeled as isotropic homogeneous bodies in numerical simulations, in reality they are neither isotropic nor homogeneous. The random grain orientation and arrangement, together with the inherent anisotropic properties of each grain, renders each joint a rather stochastic microstructure resulting in unique thermomechanical response of each joint. A grain-scale modeling approach has been proposed to assess the degree of response variability caused by the stochastic variability of the grain structure. To accomplish this, properties of both individual grains and the accommodating grain boundaries are necessary. The steady-state creep behavior of single crystal has been modeled with the Hill anisotropic potential and a Norton creep-flow rule, with model constants obtained from an experimentally-calibrated crystal-viscoplastic model presented earlier by the authors [23]. The grain boundary properties are estimated in this study from creep shear experiments conducted on selected coarse-grain solder joint test specimens. Based on the grain structure of these test specimens, obtained from EBSD images, a grain-scale finite element model of the solder joint is developed. The grain boundaries are simulated with cohesive elements having isotropic linear viscoplastic properties. The grain boundary creep model constant is determined by matching this finite element simulation result to the experiment, and then verified on another sample. The verification exercise shows reasonable agreement. Although there is a good quantitative match between experiments and simulations, given the limited number of samples, calibration with additional tests under various conditions is required for further analysis. The simulation results demonstrate that the strain heterogeneity within the solder bulk is susceptible to the grain structure, showing significant differences between samples.

This grain-scale modeling methodology enables user-friendly finite element numerical simulations of multigrain solder joints in microelectronic assemblies and facilitates parametric sensitivity studies of different grain configurations. Such a strategy provides an explicit way to quantitatively evaluate the variability between behavior of different solder joints, even those that follow identical manufacturing process and are subjected to similar loading conditions. Therefore, for a given oligocrystalline SAC solder joint, the effect of stochastic variability of grain morphology is accessible, which cannot be achieved by conventional homogeneous isotropic modeling. This capability will empower designers to numerically explore worst-case and best-case microstructural configurations (and associated stochastic variabilities in solder joint performance and design margins) under given loading conditions.

In closing, we note that, the grain structure of each individual grain is not known a priori, when modeling solder joints in electronic assemblies. Thus, it is neither efficient to model the grain structure of each individual joint explicitly nor practical to obtain the grain structure without destroying the components. Instead, the purpose of grain-level modeling is to provide a proactive capability to designers and reliability engineers to estimate the possible range of stochastic variability of observed mechanical behavior, through predictive parametric sensitivity studies of different feasible grain structures, as an effective complement to (or proxy for) physical testing. This will allow design for reliability, and significantly reduce the cost and time usually required for statistically-significant physical testing of suitably large sample sizes.

## Figures and Tables

**Table 1:** Parameters for Hill–Norton models.

A	n	F=G	H	L=M	N
9.51 × 10^−12^	3.05	1	2.75	2.24	2.15

## Data Availability

The data presented in this study are available on request from the corresponding author. At the time the project was carried out, there was no obligation to make the data publicly available.
